# Supplementary material for: Herbivory and nutrients shape grassland soil seed banks
Source: Nat Commun. 2023 Jul 4;14:3949. doi: 10.1038/s41467-023-39677-x (PMC10319882; doi:10.1038/s41467-023-39677-x)
Supplement: Supplementary file 1 — Supplementary Information [file 41467_2023_39677_MOESM1_ESM.pdf]

Supplementary file

Herbivory and nutrients shape grassland soil seed banks

Anu Eskelinen, Maria-Theresa Jessen, Hector A. Bahamonde, Jonathan D. Bakker, Elizabeth T. Borer, Maria C. Caldeira, W Stanley Harpole, Meiyu Jia, Luciola S. Lannes, Carla Nogueira, Harry Olde Venterink, Pablo L. Peri, Anita Porath-Krause, Eric W. Seabloom, Katie Schroeder, Pedro M. Tognetti, Simone-Louise E. Yasui, Risto Virtanen, and Lauren L. Sullivan

Supplementary Table S1. Site information for seven seed bank sites that participated this study. MAT; mean annual temperature in °C, MAP; mean annual precipitation in mm. See Fig. 1 for site locations in a map.

Site name	Site code	Country	Continent	Latitude	Longitude	MAT (°C)	MAP (mm)	Domestic herbivores	Vegetation type	Start of the basic NutNet experiment	Number of blocks	Seed bank sampling year	Month/timing of growing season when fertilized	How many times fertilized per year	Duration of seed bank add-on in a greenhouse	Average greenhouse temperature (°C)/humidity (%)	Dormancy break treatment	Deviations from the basic seed bank sampling protocol
Burrawan	burrawan.au	Australia	Australia	-27.7348	151.1395	18.2	643	Yes	Semiarid grassland	2008	3	2019	December/early summer	Once	Feb 2019 – Jul 2019	25 /55	None	None
Companhia das Lezirias	comp.pt	Portugal	Europe	38.8292	-8.7914	16.6	564	No	Annual grassland	2012	3	2017	September/start of growing season	Once	Feb 2018 - Feb 2019	21.6/62	Heat	None
Kilpisjärvi	kilp.fi	Finland	Europe	69.0567	20.8747	-3.3	569	No	Tundra grassland	2013	3	2017	June, July/early and mid-summer	Twice	Dec 2017 - Jan 2019	20/70	Cold	None
Cedar Creek LTER	cdcr.us	Minnesota, USA	North America	45.425	-93.2115	6.3	740	No	Tallgrass prairie	2007	5	2017	September/late summer	Once	Aug 2017 - Mar 2019	22/60	Cold	None
Smith Prairie	smith.us	Washington, USA	North America	48.2065	-122.6247	10.2	605	No	Mesic grassland	2007	3	2017	March/early spring	Once	Feb 2018 - Jan 2019	21/53	Soil disturbance	5 × 10 cm diameter round soil corers
Potrok Aike	potrok.ar	Argentina	South America	-51.916	-70.407	6.3	202	Yes	Semiarid grassland	2015	3	2017	October or November/early summer	Once	Sep 2017 - Apr 2018	20/70	None	None
Las Chilcas	chilcas.ar	Argentina	South America	-36.2755	-58.2655	15.1	955	No	Mesic grassland	2013	3	2018	April, September, December/ autumn, early spring, early summer	Three times	Feb 2019 - Dec 2019	19/80	Drought	None

Supplementary Table S2. The results of linear mixed effects models with normal errors (seed bank richness, Shannon and inverse Simpson diversity, Bray-Curtis dissimilarity between aboveground and seed bank communities, and the biomass of graminoids, forbs, and litter in aboveground communities), and generalized linear mixed effects model with a negative binomial distribution (total, graminoid and forb abundance in seed banks). Parameter significance was assessed by χ^2-tests (two-tailed). Note that graminoids included grasses, sedges and rushes but consisted mostly of grasses while forbs included forbs and legumes but consisted mostly of forbs. All models had random intercepts for blocks within sites. R2m, marginal R^2; R2c, conditional R^2. We used trigamma estimation to obtain R^2 values for negative binomial distribution. Note that differences in marginal and conditional R^2 values are caused by large between site variation in richness, abundance, and biomass values, and within site marginal R^2 values (i.e., variation explained by the treatments at individual sites) are much greater.

	χ^2	df	*P*	R2m	R2c	χ^2	df	*P*	R2m	R2c
	Seed bank richness					*Seed bank Shannon diversity*				
Herbivore exclusion	0.0269	1	0.870			0.1471	1	0.704		
Fertilization	11.8639	1	**<0.001**			16.8784	1	**<0.001**		
Fertilization + herbivore exclusion	–	–	–			–	–	–		
				0.005	0.958				0.077	0.588
	Seed bank inverse Simpson diversity					*Seed bank total abundance*				
Herbivore exclusion	0.4241	1	0.516			4.5584	1	**0.033**		
Fertilization	10.4941	1	**0.001**			10.5263	1	**0.001**		
Fertilization + herbivore exclusion	–	–	–			6.5136	1	**0.011**		
				0.068	0.426				0.012	0.896
	Seed bank graminoid abundance					*Seed bank forb abundance*				
Herbivore exclusion	1.6199	1	0.203			2.7593	1	0.097		

Fertilization	11.6840	1	**<0.001**			0.0388	1	0.844		
Fertilization + herbivore exclusion	4.9429	1	**0.026**			3.6806	1	**0.055**		
				0.024	0.786				0.009	0.862
	Dissimilarity between aboveground communities and seed banks					*Graminoid biomass in aboveground communities*				
Herbivore exclusion	1.0562	1	0.304			3.1331	1	**0.077**		
Fertilization	5.3929	1	**0.020**			4.4323	1	**0.035**		
Fertilization + herbivore exclusion	–	–	–			–	–	–		
				0.037	0.479				0.033	0.600
	Forb biomass in aboveground communities					*Litter mass in aboveground communities*				
Herbivore exclusion	0.7038	1	0.402			5.3132	1	**0.021**		
Fertilization	0.9249	1	0.336			0.7694	1	0.380		
Fertilization + herbivore exclusion	5.2650	1	**0.022**			–	–	–		
				0.023	0.805				0.045	0.418

Supplementary Table S3. Author contributions and site-level acknowledgements.

Name	Sites used in the analysis	Designed the seed bank study	Analyzed data	Contributed to the analysis	Wrote the paper	Contributed to paper writing	Performed seed bank study	Site Coordinator	Nutrient Network coordinator	Site-level acknowledgements
Anu Eskelinen	kilp.fi	x		x	x		x	x		Academy of Finland (project 297191)
Hector A. Bahamonde	potrok.ar					x	x			
Jonathan D. Bakker	smith.us					x	x	x		
Elizabeth T. Borer	cdcr.us					x		x	x	
Maria C. Caldeira	comp.pt					x	x	x		Forest Research Centre is a research unit funded by FCT
W. Stanley Harpole						x		x		
Maria-Theresa Jessen	kilp.fi					x	x			
Meiyu Jia	smith.us					x	x			National Natural Science Foundation of China (31170494); East China University of Technology Research Foundation for Career special project (DHBK2019103)
Luciola S. Lannes						x	x			Suzano Papel e Celulose for allowing long-term research on their property and for field
Carla Nogueira	comp.pt					x				
Harry Olde Venterink						x	x	x		
Pablo L. Peri	potrok.ar					x	x	x		
Anita Porath-Krause	cdcr.us					x	x			
Eric W. Seabloom	cdcr.us					x		x	x	
Katie Schroeder	cdcr.us					x	x			
Pedro M. Tognetti	chilcas.ar					x	x	x		PICT-201-1109 // UBACyT-20020170100191BA // UBACyT-20020190100212BA
Simone-Louise E. Yasui	burrowan.au					x	x			

Risto Virtanen	kilp.fi			x	x	x
Lauren Sullivan	cdcr.us	x	x	x	x	

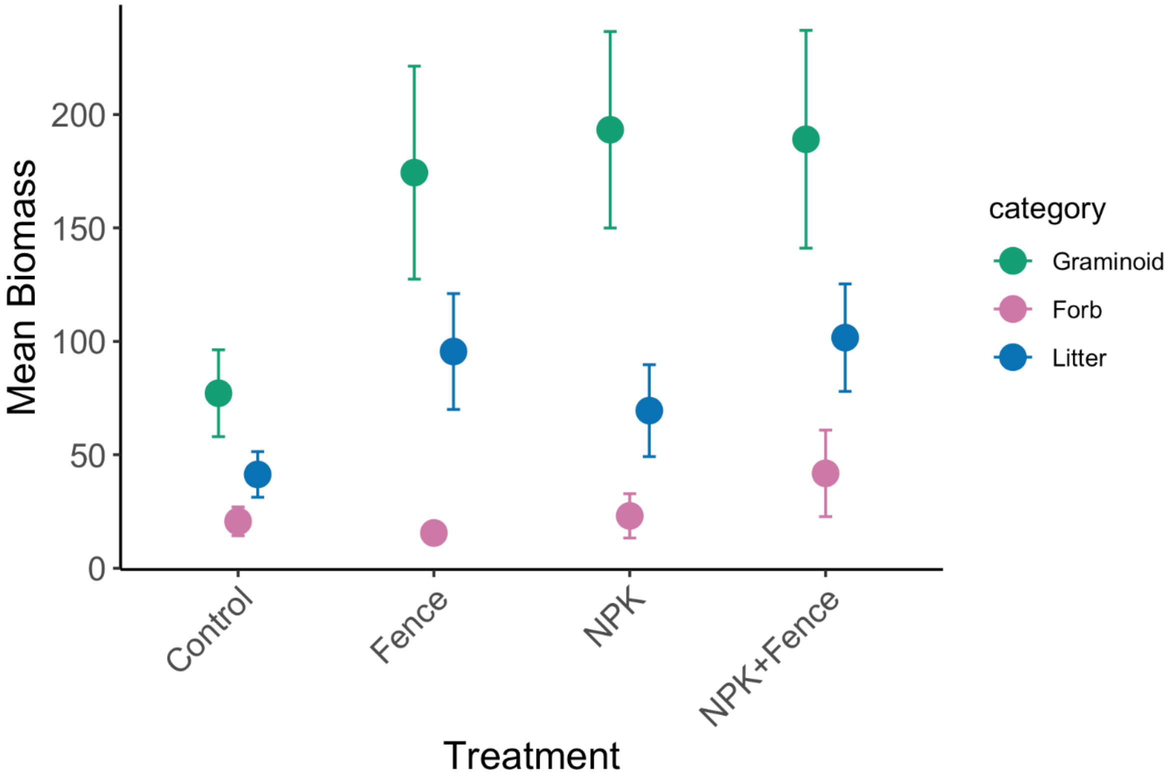

Supplementary Fig. S1. Biomass of different functional groups in aboveground communities with respect to herbivore exclusion and fertilization treatments. The biomass (g) per m^2 of graminoids, forbs, and litter in aboveground communities pooled across seven grassland sites on four continents and in different treatment combinations at the year of seed bank sampling. Graminoids include sedges and rushes but consist mostly of grasses. Forbs include legumes but consist mostly of forbs. Points represent data means and error bars represent standard error; n = 23 for quadrats from which biomass of different functional groups and litter were estimated for all treatment combinations. Fence, herbivore exclusion treatment; NPK, fertilization treatment.
